# Local methylprednisolone irrigation and tolerability after PTS-based bronchoscopic treatment for malignant central airway obstruction: a retrospective study

**DOI:** 10.3389/fmed.2026.1876205

**Published:** 2026-08-14

**Authors:** Yedan Wu, Li Zhang

**Affiliations:** Department of Respiratory & Critical Care Medicine, Chest Hospital, Tianjin University, Tianjin, China

**Keywords:** airway inflammation, bronchoscopy, local irrigation, malignant central airway obstruction, methylprednisolone, para-toluenesulfonamide

## Abstract

**Background:**

Malignant central airway obstruction (MCAO) is a severe complication of advanced thoracic malignancies. Bronchoscopic intervention combined with intralesional para-toluenesulfonamide (PTS) injection can restore airway patency, but local mucosal inflammation after treatment may cause pain, edema, and poor early tolerability. This study assessed whether local methylprednisolone irrigation after PTS-based bronchoscopic treatment improves short-term outcomes in patients with MCAO.

**Methods:**

We retrospectively analyzed 88 treatment sessions from 35 consecutive patients who underwent bronchoscopic intervention plus intralesional PTS injection between January 2024 and December 2025. According to the irrigation solution used after PTS injection, sessions were divided into methylprednisolone and normal saline groups. Outcomes at 72 h included cough score, mMRC dyspnea score, airway stenosis score, pain score, mucosal edema, and bleeding. Linear mixed-effects models and generalized estimating equations were used to account for repeated treatment sessions, and inverse probability of treatment weighting was applied to adjust for baseline differences.

**Results:**

Bronchoscopic intervention plus PTS injection significantly improved cough, dyspnea, and airway stenosis at 72 h. Compared with normal saline, methylprednisolone irrigation did not provide additional improvement in these short-term efficacy outcomes. However, methylprednisolone irrigation was associated with lower post-procedural pain and less mucosal edema, without increasing bleeding risk. These findings remained consistent after weighting, sensitivity analyses, and false discovery rate correction.

**Conclusion:**

Local methylprednisolone irrigation after PTS-based bronchoscopic treatment may improve early procedural tolerability in patients with MCAO by reducing post-procedural pain and mucosal edema, rather than enhancing short-term airway-opening efficacy. Further prospective studies are needed to confirm these findings.

## Introduction

Malignant central airway obstruction (MCAO) is a life-threatening complication of advanced thoracic malignancies, often causing severe respiratory distress, recurrent infections, and markedly reduced quality of life (1, 2). Therapeutic bronchoscopy has become the cornerstone of management, rapidly restoring airway patency through mechanical debulking, ablative techniques, or stenting and providing prompt symptom relief (3).

To enhance local tumor control, intralesional therapies have increasingly been incorporated into bronchoscopic management (4). Para-toluenesulfonamide (PTS) has emerged as a potential agent due to its reported anti-tumor effects and ability to achieve high local drug concentrations with minimal systemic toxicity (5). Previous studies have suggested that bronchoscopic PTS injection may reduce tumor burden and improve symptoms in selected patients (6). In our recent clinical experience (7), bronchoscopic ablation combined with local PTS injection also achieved durable disease control and long-term survival in a patient with adenoid cystic carcinoma and central airway stenosis, supporting the feasibility of this strategy in airway tumor management.

In clinical practice, patients undergoing bronchoscopic intervention combined with local PTS injection may develop pronounced mucosal reactions, including edema, congestion, and inflammatory exudation. These local responses may impair early recovery, reduce procedural tolerability, and potentially offset the functional benefits of airway recanalization. Therefore, strategies aimed at modulating the local inflammatory environment after treatment may be clinically relevant.

Methylprednisolone is a glucocorticoid with well-established anti-inflammatory properties (8, 9). Local administration may attenuate mucosal inflammation by reducing vascular permeability, suppressing inflammatory mediator release, and limiting tissue edema. However, the clinical impact of local methylprednisolone irrigation following PTS-based bronchoscopic treatment has not been systematically evaluated.

The present study specifically assessed both short-term efficacy (cough severity, dyspnea, and airway stenosis) and post-procedural tolerability (pain, mucosal edema, and bleeding) in patients with MCAO undergoing PTS-based bronchoscopic treatment, with adjustment for confounding by propensity score inverse probability of treatment weighting and multiple sensitivity analyses. We hypothesized that local methylprednisolone irrigation reduces post-procedural mucosal inflammation and improves short-term tolerability.

## Materials and methods

This retrospective single-center cohort study was conducted at the Department of Respiratory and Critical Care Medicine, Tianjin Chest Hospital, Tianjin University, China. We reviewed 88 treatment sessions from 35 consecutive adult patients with MCAO who underwent bronchoscopic intervention with intralesional PTS injection between January 2024 and December 2025.

As several patients received more than one intervention during the study period, each treatment session was used as the unit of analysis. Patient-level clustering was considered in all statistical models.

The study was approved by the Institutional Review Board of Tianjin Chest Hospital, Tianjin University Approval No.: 2026LW-043. The requirement for written informed consent was waived because of the retrospective design and use of de-identified clinical data. The study was conducted in accordance with the Declaration of Helsinki.

### Bronchoscopic procedures and local treatment

All procedures were performed by experienced bronchoscopists according to routine institutional practice. Additional bronchoscopic techniques were selected according to lesion characteristics, degree of airway obstruction, and procedural needs. These included mechanical debulking, cryotherapy, electrocautery, argon plasma coagulation, contact laser therapy, or their combination, as clinically indicated. Cryotherapy was performed using either a freeze–cut technique for debulking or a freeze–thaw technique for local ablation, depending on lesion characteristics and procedural goals. Electrocautery, argon plasma coagulation, and contact laser therapy were applied according to residual tumor burden, bleeding risk, and airway patency. During thermal treatment, the inspiratory oxygen fraction was reduced when necessary to minimize the risk of airway fire.

After airway patency was restored as much as possible, local PTS injection was given into the residual tumor tissue or tumor base under endobronchial ultrasound (EBUS) guidance. The commercial PTS kit contained one 5 mL vial with 1.65 g PTS and one 2 mL vial of diluent. Immediately before use, the two components were mixed at a ratio of 5:2, giving a final PTS concentration of approximately 0.33 g/mL. A bronchoscopic injection needle was used to deliver multipoint injections, usually into approximately 3–5 sites according to lesion size and morphology. At each site, a small aliquot of PTS solution was injected while gradually withdrawing the needle to facilitate intratumoral dispersion. Injection depth was controlled by real-time EBUS visualization, predetermined needle extension, and tactile resistance during needle advancement. Injection was performed only after the needle tip was confirmed to be within the tumor. The injection dose and number of injection sites were determined by lesion size, extent of airway involvement, and operator judgment.

Immediately after PTS injection, the treated area was irrigated with either methylprednisolone solution or normal saline. In the methylprednisolone group, 20 mg of methylprednisolone succinate diluted in 20 mL of 0.9% normal saline was gently instilled through the bronchoscope working channel. In the control group, the same volume of 0.9% normal saline was used. The irrigation solution was chosen by the treating bronchoscopist according to routine clinical judgment and was not randomized. Methylprednisolone irrigation was not selected on the basis of more extensive baseline tumor obstruction or more severe pre-existing airway inflammation/edema. Its main intended role was to attenuate local mucosal irritation and inflammatory reaction after PTS-based bronchoscopic treatment, including mucosal injury related to interventional techniques such as tumor debulking, electrocautery, argon plasma coagulation, cryotherapy, and contact laser therapy as well as potential local chemical irritation from limited exposure of the adjacent airway mucosa to the ethanol-containing PTS preparation during intratumoral injection. Because the mucosal inflammation and edema evaluated in this study were post-procedural local reactions, they could not be systematically graded at baseline before the intervention; therefore, potential selection bias related to operator judgment could not be completely excluded.

### Outcome assessment

Cough severity, dyspnea, and airway stenosis were assessed at baseline, immediately before the procedure, and again 72 h after the procedure. Pain, mucosal edema, and bleeding were assessed at the 72 h follow-up. Evaluations were performed by the treating bronchoscopist or by a trained respiratory physician who was not involved in the statistical analysis.

Short-term efficacy outcomes included cough severity, dyspnea, and airway stenosis. Cough severity was assessed using a visual analog scale (VAS, range 0–10), and dyspnea was assessed using the mMRC scale (range 0–4). Post-procedural tolerability outcomes included pain intensity, mucosal edema, and bleeding. Pain intensity was assessed using a numeric rating scale (NRS, range 0–10). Airway stenosis, mucosal edema, and bleeding were evaluated bronchoscopically using predefined ordinal scales. Airway stenosis was graded as follows: 0, no significant stenosis; 1, < 25% luminal narrowing; 2, 26–0% narrowing; 3, 51%−75% narrowing; and 4, >75% narrowing or near-complete occlusion. Mucosal edema was graded as 0, none; 1, mild; 2, moderate; and 3, severe (10). Bleeding was graded as 0, none; 1, mild bleeding controlled by suction alone; 2, moderate bleeding requiring topical epinephrine spraying; and 3, severe bleeding requiring additional bronchoscopic intervention or escalation of care (11).

### Statistical analysis

Continuous variables are reported as mean ± standard deviation or median with interquartile range, and categorical variables as counts and percentages. Baseline characteristics were compared between the methylprednisolone and normal saline groups using the independent *t*-test, Wilcoxon rank-sum test, χ^2^ test, or Fisher's exact test, as appropriate. Standardized mean differences were also calculated to quantify baseline imbalance between groups, with values < 0.10 considered indicative of acceptable balance.

Changes in cough score, mMRC dyspnea score, and airway stenosis score from baseline to 72 h were analyzed using linear mixed-effects models with a random intercept for each patient. The models included time, treatment group, the time × group interaction, and combined bronchoscopic intervention. Pain, mucosal edema, and bleeding at 72 h were analyzed using generalized estimating equations with an exchangeable correlation structure and robust standard errors.

To reduce confounding from non-random treatment allocation, inverse probability of treatment weighting was performed using propensity scores. The propensity model included age, sex, COPD status, combined bronchoscopic intervention, and baseline cough, mMRC, and airway stenosis scores. COPD status was included as a clinically relevant baseline covariate because it may affect respiratory symptom assessment, particularly cough severity and mMRC dyspnea score, and may influence clinicians' decisions regarding peri-procedural corticosteroid use. Given the limited number of COPD cases in this cohort, the study was not designed to evaluate the independent effect of COPD on post-procedural pain, mucosal edema, bleeding, or mucosal healing. Stabilized weights were used, and covariate balance was assessed by standardized mean differences, with values < 0.10 considered acceptable.

Model assumptions were checked before analysis. The Benjamini–Hochberg procedure was applied separately to the efficacy and tolerability outcomes to control the false discovery rate. All tests were two-sided, and *P* < 0.05 was considered statistically significant. Analyses were performed using R software version 4.3.2.

## Results

### Baseline characteristics

A total of 88 treatment sessions from 35 patients with MCAO were included. Of these, 38 sessions received normal saline irrigation and 50 received methylprednisolone irrigation after PTS injection (Table 1). As shown in the revised Table 1, COPD status and the use of combined bronchoscopic intervention were generally similar between the normal saline and methylprednisolone groups. However, the normal saline group had greater baseline respiratory symptom burden, with higher pre-treatment cough and mMRC dyspnea scores, as well as a tendency toward more severe airway stenosis. These baseline differences were quantified using *P* values and standardized mean differences and were addressed in the subsequent IPTW-adjusted analyses.

**Table 1:** Baseline characteristics of treatment sessions.

Variable	Overall (*n* = 88)	NS group (*n* = 38)	GC group (*n* = 50)	*P* value	SMD
COPD, *n* (%)				0.600	0.165
No	83 (94.3)	35 (92.1)	48 (96.0)		
Yes	5 (5.7)	3 (7.9)	2 (4.0)		
Combined bronchoscopic intervention, *n* (%)				0.500	0.133
No	43 (48.9)	20 (52.6)	23 (46.0)		
Yes	45 (51.1)	18 (47.4)	27 (54.0)		
Pre-treatment mMRC score	2.11 ± 0.60/2.00 (2.00–2.00)	2.34 ± 0.48/2.00 (2.00–3.00)	1.94 ± 0.62/2.00 (2.00–2.00)	< 0.001	0.409
Pre-treatment cough score	4.91 ± 1.45/5.00 (4.00–6.00)	5.55 ± 1.29/5.50 (4.00–6.00)	4.42 ± 1.39/4.00 (3.00– 5.00)	< 0.001	0.448
Pre-treatment airway stenosis score	2.72 ± 1.00/2.00 (2.00–4.00)	3.04 ± 1.04/3.50 (2.00–4.00)	2.54 ± 0.94/2.00 (2.00–3.00)	0.052	0.278

Data are presented as *n* (%) or mean ± SD / median (IQR). *P* values were calculated using Fisher's exact test, Pearson's chi-square test, or the Wilcoxon rank-sum test, as appropriate. SMDs are presented as absolute standardized mean differences before weighting. NS, normal saline; COPD, chronic obstructive pulmonary disease; mMRC, modified Medical Research Council; SMD, standardized mean difference.

**Figure 1:**
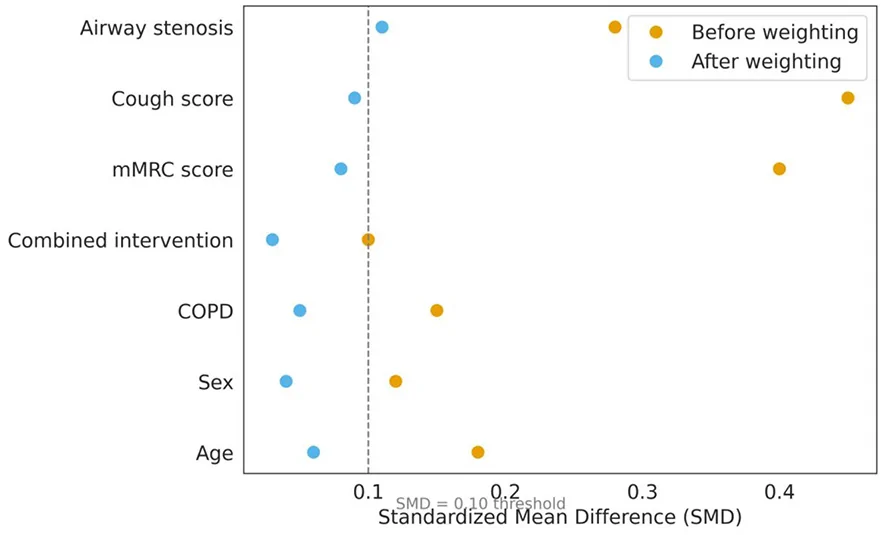
Covariate balance before and after propensity score weighting.

After stabilized inverse probability of treatment weighting, covariate balance improved substantially. Most standardized mean differences were below 0.10, with only mild residual imbalance in pre-treatment airway stenosis score (Figure 1 and Supplementary Table S1).

Additional procedural characteristics are summarized descriptively in Supplementary Table S4. Tumor histology was summarized at the patient level in Supplementary Table S5 to avoid repeated counting of patients who underwent more than one treatment session.

### Short-term efficacy outcomes

Bronchoscopic intervention combined with intralesional PTS injection was associated with clear improvement at 72 h. Cough score decreased by 2.71 points (95% CI −3.14 to −2.28, *P* < 0.001), mMRC dyspnea score by 1.00 point (95% CI −1.06 to −0.94, *P* < 0.001), and airway stenosis score by 1.54 points (95% CI −1.93 to −1.14, *P* < 0.001) (Table 2).

**Table 2:** Mixed-effects model results for short-term efficacy outcomes.

Outcome	Variable	Estimate (95% CI)	*P* value
Cough score	Time (post vs. pre)	−2.71 (−3.14 to −2.28)	< 0.001
	Group (GC vs. NS)	−1.15 (−1.72 to −0.57)	< 0.001
	Time × Group	−0.07 (−0.64 to 0.50)	0.810
	Combined intervention	0.58 (0.15 to 1.01)	0.009
mMRC score	Time (post vs. pre)	−1.00 (−1.06 to −0.94)	< 0.001
	Group (GC vs. NS)	−0.18 (−0.58 to 0.22)	0.363
	Time × Group	−0.02 (−0.10 to 0.06)	0.632
	Combined intervention	0.14 (0.04 to 0.24)	0.005
Airway stenosis score	Time (post vs. pre)	−1.54 (−1.93 to −1.14)	< 0.001
	Group (GC vs. NS)	−0.45 (−0.94 to 0.04)	0.072
	Time × Group	0.43 (−0.06 to 0.92)	0.087
	Combined intervention	0.58 (0.22 to 0.94)	0.002

Estimates represent regression coefficients with 95% confidence intervals.

Models included time (pre- vs. post-treatment), treatment group, their interaction, and combined bronchoscopic intervention as a fixed effect, with patient-level random intercepts.

Methylprednisolone irrigation did not provide additional improvement in these efficacy outcomes. The time × group interaction was not significant for cough score (β = −0.07, 95% CI −0.64 to 0.50, *P* = 0.810), mMRC dyspnea score (β = −0.02, 95% CI −0.10 to 0.06, *P* = 0.632), or airway stenosis score (β = 0.43, 95% CI −0.06 to 0.92, *P* = 0.087). The group differences observed in some models were therefore more likely related to baseline imbalance than to an additional treatment effect of methylprednisolone.

### Treatment tolerability and local reactions

At 72 h, methylprednisolone irrigation was associated with better post-procedural tolerability. Compared with normal saline, it was associated with lower pain scores (β = −1.13, 95% CI −2.04 to −0.22, *P* = 0.015) and less mucosal edema (β = −0.85, 95% CI −1.29 to −0.41, *P* < 0.001) (Table 3). Bleeding scores did not differ significantly between groups (β = −0.25, 95% CI −1.02 to 0.53, *P* = 0.537).

**Table 3:** GEE analysis for post-procedural tolerability and local reactions.

Outcome	Variable	Estimate (95% CI)	*P* value
Pain score	Group (GC vs. NS)	−1.13 (−2.04 to −0.22)	0.015
	Combined intervention	0.84 (0.17 to 1.51)	0.014
Mucosal edema	Group (GC vs. NS)	−0.85 (−1.29 to −0.41)	< 0.001
	Combined intervention	0.40 (0.07 to 0.73)	0.018
Bleeding	Group (GC vs. NS)	−0.25 (−1.02 to 0.53)	0.537
	Combined intervention	0.78 (0.26 to 1.31)	0.004

Estimates represent regression coefficients with 95% confidence intervals.

GEE models used an exchangeable correlation structure with robust standard errors to account for within-patient correlation.

**Figure 2:**
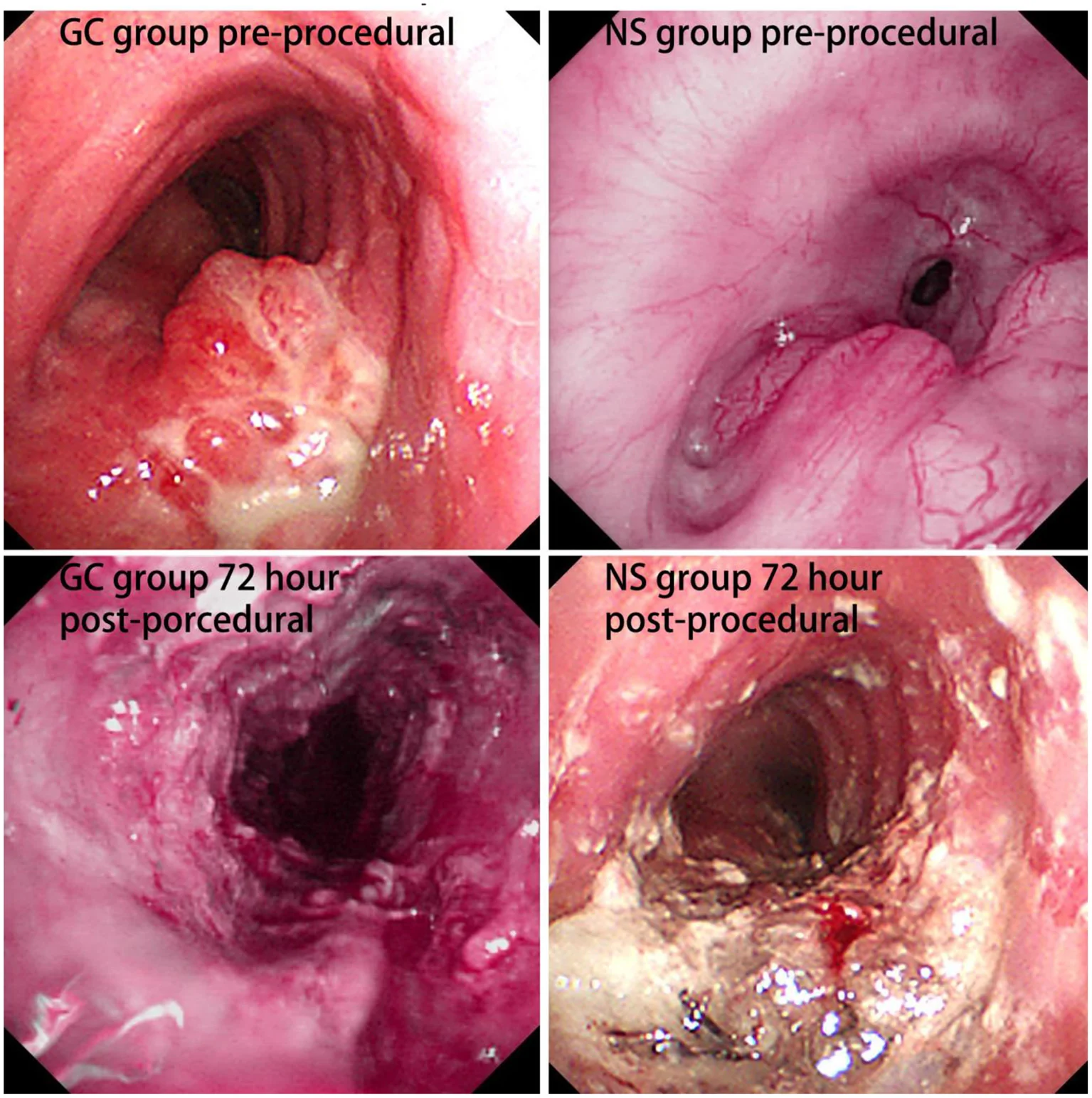
Representative bronchoscopic images illustrating airway patency and local mucosal appearance before and after PTS-based bronchoscopic treatment. Representative 72 h post-procedural bronchoscopic view in the normal saline group showing improved airway patency after bronchoscopic intervention and intralesional PTS injection, with more evident mucosal edema at the treated site than in the methylprednisolone group.

These findings were consistent in the IPTW-adjusted models, patient-level analyses, and analyses restricted to the first treatment session per patient (Supplementary Table S2). The main tolerability findings also remained significant after Benjamini–Hochberg false discovery rate correction (Supplementary Table S3). Representative bronchoscopic images are shown in Figure 2 to illustrate airway patency changes after PTS-based bronchoscopic treatment and the post-procedural mucosal appearance in the normal saline and methylprednisolone groups. The overall pattern of treatment effects is shown in Supplementary Figure S1.

### Additional observations

Combined bronchoscopic intervention was associated with higher symptom burden and more local reactions in the models (Tables 2, 3). This likely reflects more severe baseline disease and greater procedural complexity rather than a direct adverse effect of combined treatment. The overall pattern of treatment effects is shown in Supplementary Figure S1.

## Discussion

In this retrospective study, local methylprednisolone irrigation after PTS-based bronchoscopic treatment was not associated with additional improvement in short-term efficacy outcomes, including cough, dyspnea, and airway stenosis. Its main benefit was seen in post-procedural tolerability, with lower pain and mucosal edema scores at 72 h and no increase in bleeding. These findings suggest that local methylprednisolone mainly acts as an anti-inflammatory adjunct rather than as a treatment that enhances immediate airway recanalization.

The improvement in cough, dyspnea, and airway stenosis observed in both groups is consistent with the expected effect of therapeutic bronchoscopy in MCAO. Previous studies have shown that endoscopic or bronchoscopic management of MCAO can provide clinically meaningful benefits in dyspnea, functional status, quality of life, and selected survival-related outcomes (12, 13). Stratakos et al. (12) reported survival and quality-of-life benefits after endoscopic management of malignant central airway obstruction, supporting the clinical relevance of restoring central airway patency in this population. However, technical success does not always translate into equivalent clinical benefit, as patient condition, obstruction pattern, tumor burden, distal airway patency, and procedural complexity may all influence outcomes (14). Our findings are consistent with this broader evidence base: the major short-term improvement in airway stenosis and respiratory symptoms was most likely driven by bronchoscopic recanalization itself, including mechanical debulking, ablative techniques, balloon dilation, stent placement, or their combination.

**P**TS-based local therapy provides an additional tumor-directed strategy in this setting. Previous clinical studies have reported that intratumoral PTS injection may reduce tumor burden and improve airway obstruction in patients with severe malignant airway obstruction, including non-small cell lung cancer and pulmonary adenoid cystic carcinoma (4, 5). Preclinical work has also suggested antitumor activity of PTS in lung cancer xenograft models (15). These studies mainly focused on tumor response, airway obstruction, and disease control. By contrast, the present study addressed a different and more immediate clinical question: whether local corticosteroid irrigation after PTS-based bronchoscopic treatment can improve early post-procedural tolerability. Therefore, our results should be viewed as complementary to previous PTS studies rather than as evidence that methylprednisolone enhances the antitumor effect of PTS.

The lack of additional improvement in cough, dyspnea, or airway stenosis with methylprednisolone irrigation is clinically plausible. Immediate airway-opening efficacy after therapeutic bronchoscopy depends mainly on mechanical and endoscopic factors, including the extent of tumor removal, relief of fixed obstruction, airway dilation, and stent placement when required. Corticosteroids do not remove intraluminal tumor, reverse extrinsic compression, or immediately enlarge a mechanically obstructed airway. Therefore, the absence of a significant time-by-group interaction for the efficacy outcomes supports a restrained interpretation: methylprednisolone may modulate the local inflammatory response after treatment, but it should not be considered an airway recanalization therapy.

The reduction in pain and mucosal edema is also biologically reasonable. Bronchoscopic manipulation, thermal ablation, cryotherapy, balloon dilation, stent placement, and local injection can all cause acute mucosal injury. In patients receiving intralesional PTS, local tumor necrosis and inflammatory exudation may further contribute to short-term mucosal swelling and discomfort. Although direct evidence regarding methylprednisolone irrigation after PTS-based bronchoscopic treatment is limited, previous bronchoscopic experience has suggested that locally delivered corticosteroids may help reduce airway mucosal edema in selected airway injury or stenosis settings (16). Our study extends this concept to malignant airway intervention, but the interpretation should remain cautious because the present data are clinical and observational rather than mechanistic.

From a safety perspective, the absence of increased bleeding after local methylprednisolone irrigation is reassuring. Bleeding and other procedure-related complications are important considerations in therapeutic bronchoscopy for MCAO, particularly in patients requiring multimodality intervention or extensive tumor manipulation. In the present cohort, local methylprednisolone irrigation was not associated with a higher bleeding score at 72 h. This finding supports the short-term tolerability of this approach, but it does not exclude delayed effects on mucosal healing, infection, restenosis, or repeated intervention, which would require longer follow-up.

The association between combined bronchoscopic intervention and greater symptom burden or stronger local reactions should also be interpreted carefully. Patients requiring multiple bronchoscopic techniques usually have more complex obstruction, greater tumor burden, more severe airway narrowing, or a need for more extensive mucosal manipulation. Therefore, combined intervention in our models is more likely a marker of disease complexity and procedural intensity than an independent cause of poor tolerability. This interpretation is consistent with the broader interventional bronchoscopy literature, in which clinical outcomes are influenced not only by technical success but also by baseline disease severity, lesion characteristics, and patient selection (14).

This study has several clinical implications. First, early post-procedural tolerability should be considered a distinct endpoint in bronchoscopic treatment for MCAO. Technical success and airway patency remain essential, but pain, edema, cough, and short-term comfort also affect early recovery and patient experience. Second, local anti-inflammatory treatment may be useful as an adjunct in selected patients at risk of prominent mucosal reaction after PTS-based intervention. Third, the present findings do not support using methylprednisolone irrigation with the expectation of improving immediate airway-opening efficacy. Its potential value appears to lie in reducing local inflammatory reactions after the airway has been mechanically reopened.

Several limitations should be acknowledged. This was a single-center retrospective study with a modest sample size, and some patients contributed more than one treatment session; although patient-level clustering and propensity score weighting were used, residual confounding cannot be fully excluded. In addition, the choice of methylprednisolone vs. normal saline irrigation was not randomized, and procedural heterogeneity may not have been completely captured by the combined bronchoscopic intervention variable, despite additional procedural details being extracted and summarized. Finally, the follow-up period was limited to 72 h, so longer-term outcomes such as restenosis, repeated intervention, quality of life, mucosal healing, infection, and survival could not be assessed; mucosal edema and bleeding were also evaluated using semi-quantitative bronchoscopic scales, which may introduce observer variability.

Taken together, local methylprednisolone irrigation appears to have a supportive role after PTS-based bronchoscopic treatment. It may help attenuate early local inflammatory reactions and improve short-term procedural tolerability, while the main airway-opening effect remains attributable to therapeutic bronchoscopy and local tumor-directed intervention. Prospective studies with larger sample sizes, standardized bronchoscopic assessment, longer follow-up, and inflammatory biomarker evaluation are needed to confirm these findings and define which patients are most likely to benefit.

## Conclusions

Local methylprednisolone irrigation after PTS-based bronchoscopic treatment may serve as a local anti-inflammatory adjunct to improve early procedural tolerability in patients with MCAO, rather than enhancing short-term airway-opening efficacy.

## Data Availability

The original contributions presented in the study are included in the article/Supplementary material, further inquiries can be directed to the corresponding author.
