# Supplementary material for: Local methylprednisolone irrigation and tolerability after PTS-based bronchoscopic treatment for malignant central airway obstruction: a retrospective study
Source: Front Med (Lausanne). 2026 Aug 14;13:1876205. doi: 10.3389/fmed.2026.1876205 (PMC13522201; doi:10.3389/fmed.2026.1876205)
Supplement: Supplementary file 1 [file Supplementary_File_1.docx]

Effect of methylprednisolone irrigation versus normal saline on short-term outcomes. Estimates are regression coefficients with 95% confidence intervals from the primary models (linear mixed-effects models for efficacy outcomes; GEE for tolerability outcomes). P values are shown on the right.
