# Supplementary material for: Local methylprednisolone irrigation and tolerability after PTS-based bronchoscopic treatment for malignant central airway obstruction: a retrospective study
Source: Front Med (Lausanne). 2026 Aug 14;13:1876205. doi: 10.3389/fmed.2026.1876205 (PMC13522201; doi:10.3389/fmed.2026.1876205)
Supplement: Supplementary file 2 [file Table_1.docx]

Table S1. Covariate balance before and after propensity score weighting

| Variable | SMD (Before) | SMD (After) |
| --- | --- | --- |
| Age | 0.18 | 0.06 |
| Sex | 0.12 | 0.04 |
| COPD | 0.15 | 0.05 |
| Combined intervention | 0.10 | 0.03 |
| mMRC | 0.40 | 0.08 |
| Cough score | 0.45 | 0.09 |
| Airway stenosis | 0.28 | 0.11 |

An absolute SMD < 0.10 was considered indicative of acceptable balance.
