# Supplementary material for: Local methylprednisolone irrigation and tolerability after PTS-based bronchoscopic treatment for malignant central airway obstruction: a retrospective study
Source: Front Med (Lausanne). 2026 Aug 14;13:1876205. doi: 10.3389/fmed.2026.1876205 (PMC13522201; doi:10.3389/fmed.2026.1876205)
Supplement: Supplementary file 3 [file Table_2.docx]

Table S2. Sensitivity analyses for post-procedural outcomes

| Outcome | Analysis | Estimate (95% CI) | *P* value |
| --- | --- | --- | --- |
| Pain score | Primary model (GEE) | -1.13 (-2.04 to -0.22) | 0.015 |
|  | IPTW model | -0.95 (-1.80 to -0.10) | 0.028 |
|  | Patient-level analysis | -1.05 (-1.90 to -0.20) | 0.020 |
|  | Excluding repeated sessions | -1.00 (-1.85 to -0.15) | 0.022 |
| Mucosal edema | Primary model (GEE) | -0.85 (-1.29 to -0.41) | <0.001 |
|  | IPTW model | -0.72 (-1.20 to -0.25) | 0.003 |
|  | Patient-level analysis | -0.78 (-1.25 to -0.30) | 0.002 |
| Bleeding | Primary model (GEE) | -0.25 (-1.02 to 0.53) | 0.537 |
|  | IPTW model | -0.18(-0.90 to 0.55) | 0.620 |
|  | Patient-level analysis | -0.20 (-0.95 to 0.50) | 0.580 |

Estimates represent the effect of methylprednisolone irrigation (GC vs NS).

All analyses accounted for within-patient correlation where applicable.
