# Supplementary material for: Local methylprednisolone irrigation and tolerability after PTS-based bronchoscopic treatment for malignant central airway obstruction: a retrospective study
Source: Front Med (Lausanne). 2026 Aug 14;13:1876205. doi: 10.3389/fmed.2026.1876205 (PMC13522201; doi:10.3389/fmed.2026.1876205)
Supplement: Supplementary file 4 [file Table_3.docx]

Table S3. Benjamini–Hochberg false discovery rate (FDR) adjustment for multiple comparisons within each outcome domain

| Outcome domain | Specific outcome | Primary GEE / LMM model *P* value | FDR-adjusted q value |
| --- | --- | --- | --- |
| Short-term efficacy (LMM time × group interaction) | Cough score | 0.7000 | 0.7000 |
|  | mMRC dyspnea score | 0.6771 | 0.7000 |
|  | Airway stenosis score | 0.0871 | 0.2613 |
| Tolerability (GEE group difference) | Pain score | 0.015 | 0.015 |
|  | Mucosal edema score | <0.001 | <0.001 |
|  | Bleeding score | 0.537 | 0.537 |

FDR-adjusted q-values were calculated separately within the short-term efficacy domain (LMM time × group interaction terms) and the tolerability domain (GEE group differences) using the Benjamini–Hochberg procedure to control the false discovery rate at 5%. Primary model P-values correspond to the results presented in Table 2 (efficacy) and Table 3 (tolerability).
