# Supplementary material for: Local methylprednisolone irrigation and tolerability after PTS-based bronchoscopic treatment for malignant central airway obstruction: a retrospective study
Source: Front Med (Lausanne). 2026 Aug 14;13:1876205. doi: 10.3389/fmed.2026.1876205 (PMC13522201; doi:10.3389/fmed.2026.1876205)
Supplement: Supplementary file 5 [file Table_4.docx]

Table S4. Procedural characteristics of treatment sessions

| Variable | Overall (n = 88) | NS group (n = 38) | GC group (n = 50) |
| --- | --- | --- | --- |
| Mechanical debulking, n (%) | 51(57.95) | 20(52.63) | 31(62) |
| Cryotherapy, n (%) | 44(50) | 19(50) | 25(50) |
| Electrocautery, n (%) | 37(42.04) | 13(34.21) | 24(48) |
| Argon plasma coagulation, n (%) | 21(23.86) | 10(26.31) | 11(22) |
| Contact laser therapy, n (%) | 2(2.27) | 2(5.26) | 0(0) |

Data are presented as n (%). Procedural variables were summarized descriptively at the treatment-session level.
