# Supplementary material for: Local methylprednisolone irrigation and tolerability after PTS-based bronchoscopic treatment for malignant central airway obstruction: a retrospective study
Source: Front Med (Lausanne). 2026 Aug 14;13:1876205. doi: 10.3389/fmed.2026.1876205 (PMC13522201; doi:10.3389/fmed.2026.1876205)
Supplement: Supplementary file 6 [file Table_5.docx]

Table S5. Tumor histology of included patients

| Tumor histology | Overall patients (n = 35), n (%) |
| --- | --- |
| Squamous cell carcinoma | 23 (65.7) |
| Adenocarcinoma | 7 (20) |
| Small-cell lung cancer | 2 (5.71) |
| Adenoid cystic carcinoma | 1 (2.86) |
| Malignant mesenchymal tumor | 1 (2.86) |
| Metastatic tumor involving the central airway | 1 (2.86) |

Data are presented as n (%). Tumor histology was summarized at the patient level to avoid repeated counting of patients who contributed more than one treatment session.
